# Efficacy of Simvastatin in Inhibiting Bone Resorption and Promoting Healing in Delayed Tooth Avulsion: A Case Series

**DOI:** 10.7759/cureus.79139

**Published:** 2025-02-17

**Authors:** Rajesh Kumar, Supraja N Atluri, Alekhya Achanta, Chittaranjan Bogishetty, Tejaswini R Chunduri, Tejaswini PSS, Ramakrishna Ravi

**Affiliations:** 1 Paediatric Dentistry, Malla Reddy Institute of Dental Sciences, Hyderabad, IND; 2 Prosthodontics, Malla Reddy Dental College for Women, Hyderabad, IND; 3 Epidemiology, Mississippi State Department of Health, Ridgeland, USA; 4 Oral and Maxillofacial Surgery, Malla Reddy Institute of Dental Sciences, Hyderabad, IND; 5 Conservative Dentistry and Endodontics, Malla Reddy Institute of Dental Sciences, Hyderabad, IND

**Keywords:** avulsion, delayed reimplantation, hydroxyapatite, prf, simvastatin

## Abstract

An avulsion is a severe dental injury characterized by the complete displacement of a tooth from its socket, often resulting in a compromised prognosis. One of the key factors influencing the success of reimplantation is the extraoral dry time, which refers to the duration the tooth remains outside of the socket. Prolonged dry time significantly impairs the viability of the periodontal ligament cells, crucial for successful healing and reattachment. Despite various protocols and treatment strategies developed to manage avulsed teeth, no single approach addresses all treatment needs effectively, particularly in cases of delayed reimplantation.

Simvastatin, an anti-lipidemic drug, has demonstrated pleiotropic effects that extend beyond cholesterol lowering. These effects include anti-inflammatory properties, promotion of bone regeneration, and enhancement of periodontal ligament cell survival. Such actions suggest that simvastatin may have a beneficial role in improving outcomes following the delayed reimplantation of avulsed teeth.

This case series proposes the use of simvastatin as an adjunctive treatment for avulsed teeth along with platelet-rich fibrin and hydroxyapatite, particularly in situations where reimplantation is delayed. By mitigating inflammation and stimulating tissue regeneration, simvastatin may help counteract the damage caused by prolonged extraoral dry time. Its potential to promote periodontal ligament cell survival and enhance healing processes could improve the prognosis of reimplantation, even in cases where traditional treatment alone would be less effective.

Given these promising properties, simvastatin may represent a valuable addition to the treatment protocol for avulsed teeth with extended dry times. However, further clinical studies and trials are necessary to validate its efficacy and establish a clear role in the management of delayed reimplantation.

## Introduction

The incidence of traumatic injuries to anterior teeth in children is relatively higher than in adults [1,2]. The presence of spongy bone and incomplete root formation increases the incidence of avulsion in children [3]. The maxillary incisor is the most commonly affected tooth, with increased overjet and incompetent lips being predisposing factors for its incidence [4].

Reimplantation of avulsed teeth is the most preferred treatment modality. The success of a reimplanted tooth depends on age, antibiotics used, extraoral dry time, splinting, storage medium, type of dressing, and root formation [5-7]. Extraoral time and time-lapse from avulsion to reimplantation are the most important factors that determine the viability of cells [8,9]. Time-dependent factors cause irreversible damage to various components of the periodontium and can be considered the perpetuating factor for the poor prognosis of various treatment modalities [10]. To minimize inflammation from the necrotic periodontal cells, the surface of the teeth should be devoid of necrotic tissue. Hence, healing in reimplanted teeth is considerably dependent on the remnant tissue present on the alveolar side [7]. With the minimal tissue present, to enhance healing, a medicament that exerts anti-inflammatory properties, promotes differentiation of mesenchymal stem cells, and has antimicrobial activity is necessary for a successful outcome in teeth reimplantation. The efficacy of various drugs with the above properties has been evaluated in treating re-implanted teeth.

Simvastatin is one of the drugs possessing these properties and is indicated in treating hyperlipidemia. It exerts pleiotropic properties, such as anti-inflammatory, anti-thrombotic, and antimicrobial properties, promoting cell differentiation and osteogenic potential. The anti-inflammatory property is required to prevent inflammatory resorption, the antimicrobial property helps prevent secondary infections, the cell differentiation-inducing potential helps enhance the cell division and differentiation of mesenchymal stem cells on the alveolar side, and, lastly, bone formation [11]. Hence, simvastatin can be a medicament for preventing root as well as bone resorption after reimplantation. In this case series, we successfully prevented the above in reimplanted teeth with increased dry time by incorporating simvastatin along with other modalities.

## Case presentation

Case 1

**Figure 1:**
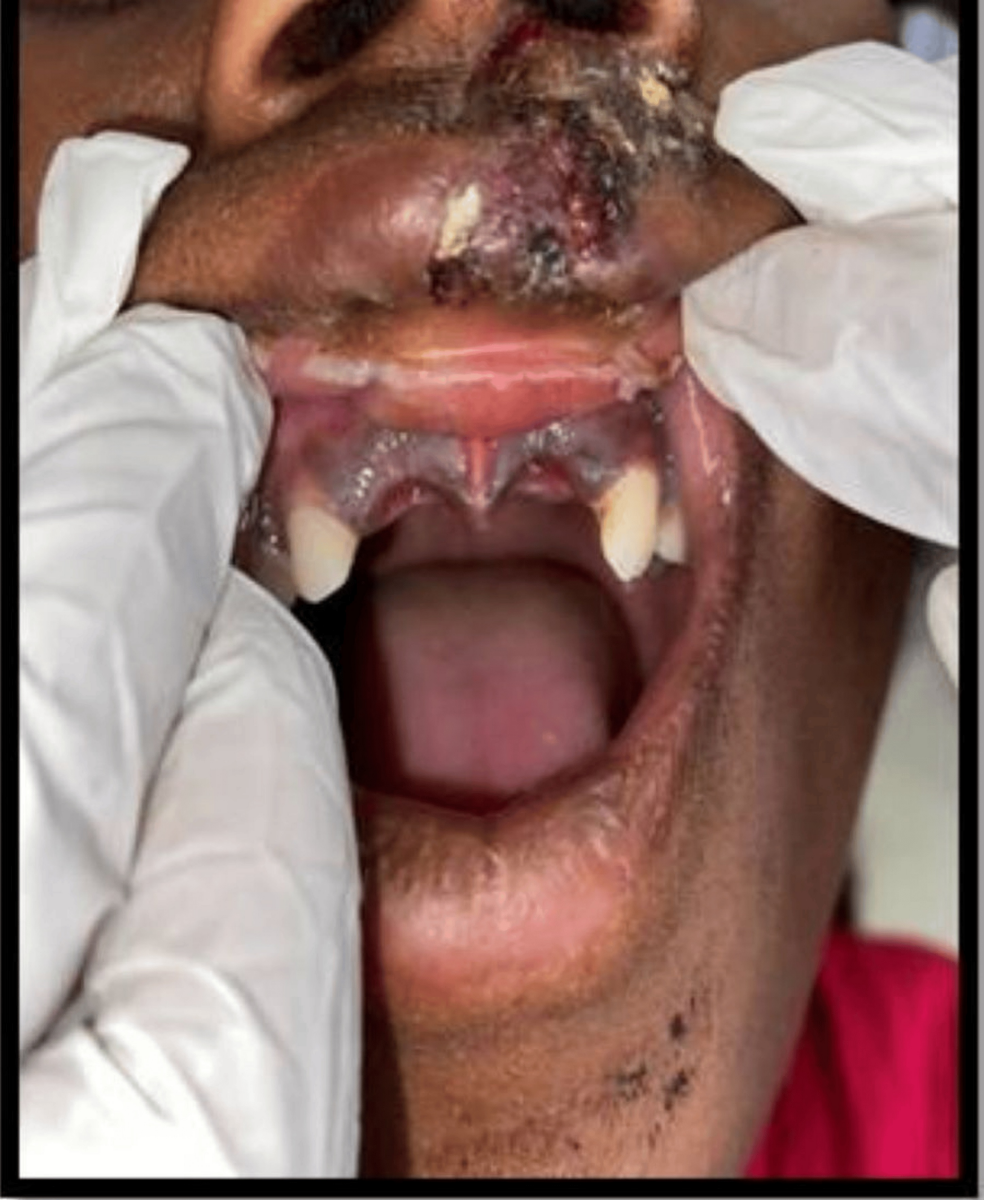
Preoperative image showing missing 11 and 21 following avulsion.

A 12-year-old girl presented to the Department of Pediatric and Preventive Dentistry seeking care for the loss of her upper two front teeth. The teeth were brought to the department in dry condition 46 hours after the accident. Medical history was non-contributory. No concomitant systemic diseases were reported by the patient’s parents. Extraoral examination revealed healing abrasions along the nasal bridge and philtrum. Intraoral examination revealed that the maxillary right and left permanent central incisor (teeth 11 and 21) were avulsed (Figure 1).

**Figure 2:**
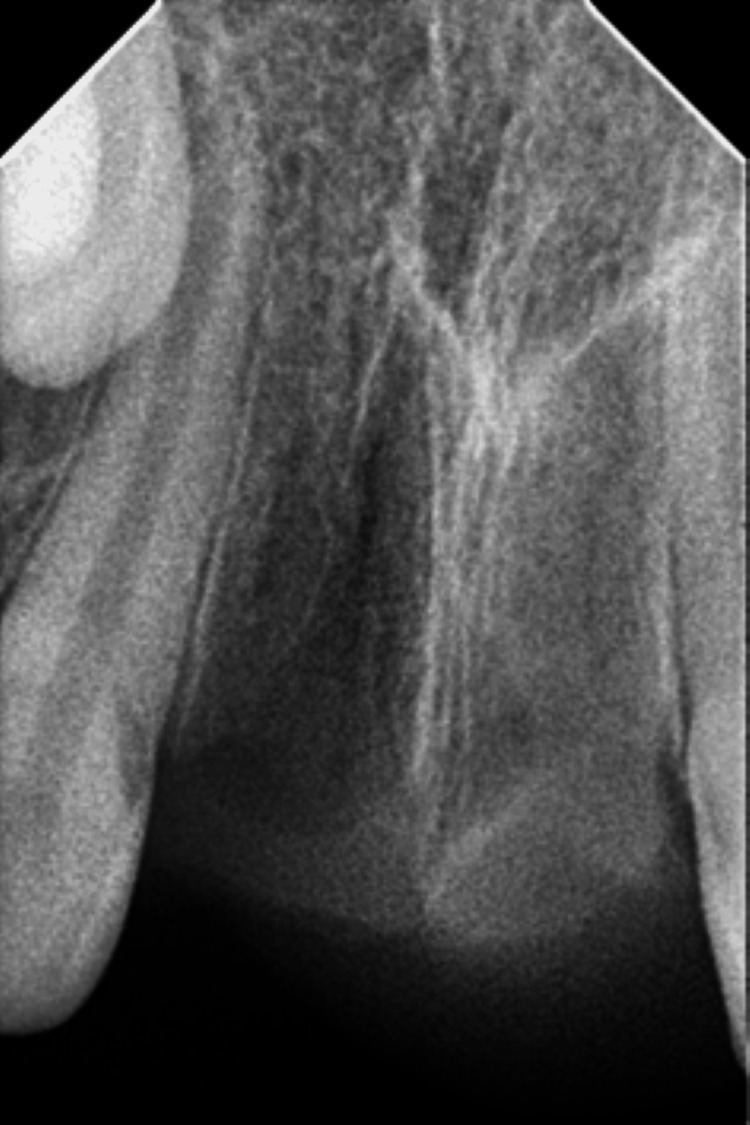
Preoperative radiograph showing the empty sockets 11 and 21 following tooth avulsion.

On inspection and palpation of the anterior maxillary segment, a dentoalveolar fracture was ruled out. Radiovisiography revealed an empty alveolar socket in the 11 and 21 regions. There were no other injuries or fractures of the adjacent teeth and associated alveolar structures (Figure 2).

**Figure 3:**
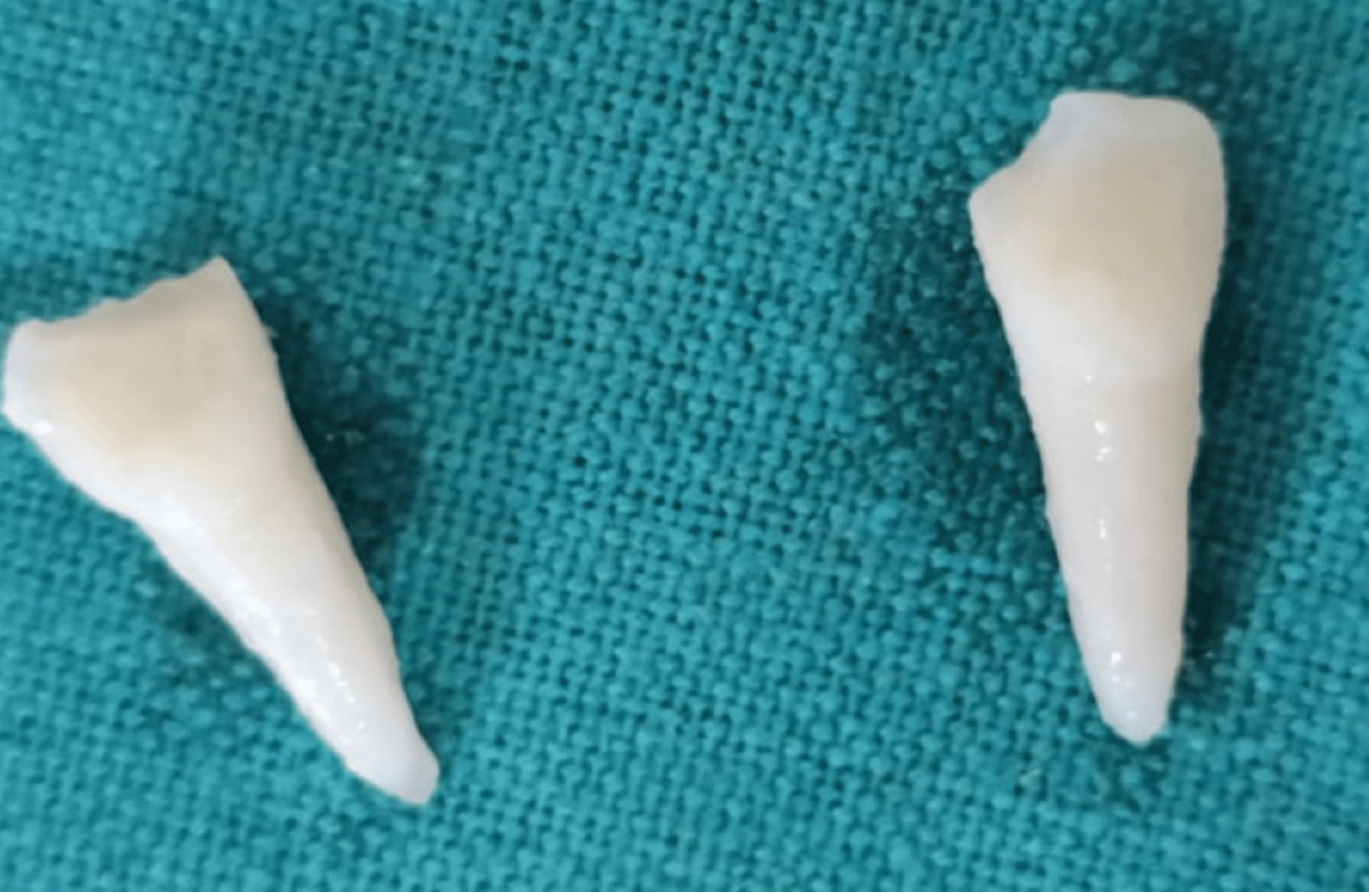
Avulsed teeth root surfaces after cleansing with acidulated phosphate fluoride.

The patient’s parents were informed about the plausible complications associated with replantation of the teeth. After obtaining informed consent, it was decided to reposition and replant the avulsed teeth. The root surface of the teeth was curetted to remove any coagulum, granulation tissue, or pathologic tissue, followed by irrigation with physiologic saline solution and treatment with 1.23% acidulated phosphate fluoride (APF) gel for five minutes (Figure 3).

**Figure 4:**
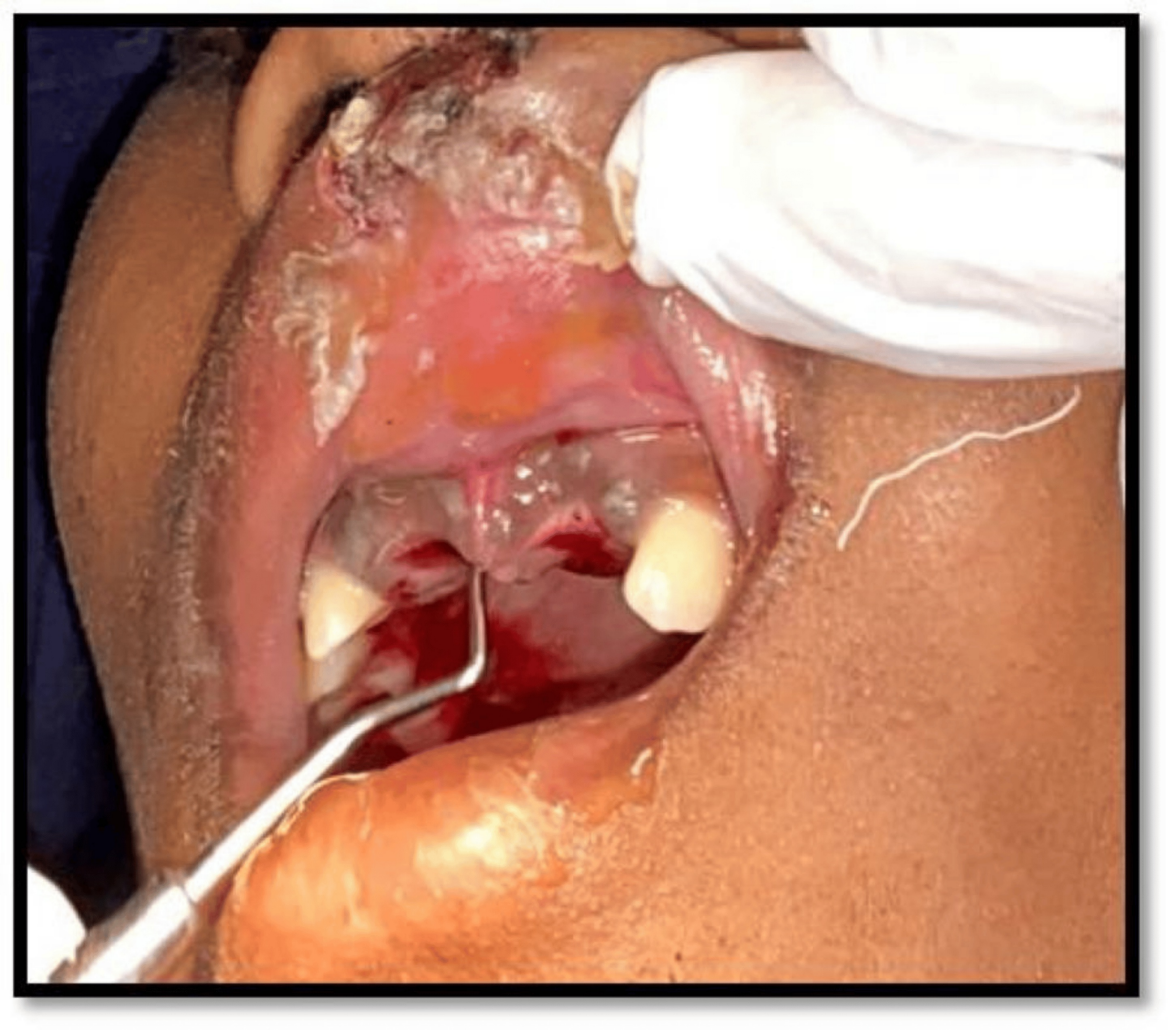
Socket debridement.

The teeth socket was gently debrided and rinsed with saline solution under local anesthesia using 2% lignocaine HCl (Figure 4).

**Figure 5:**
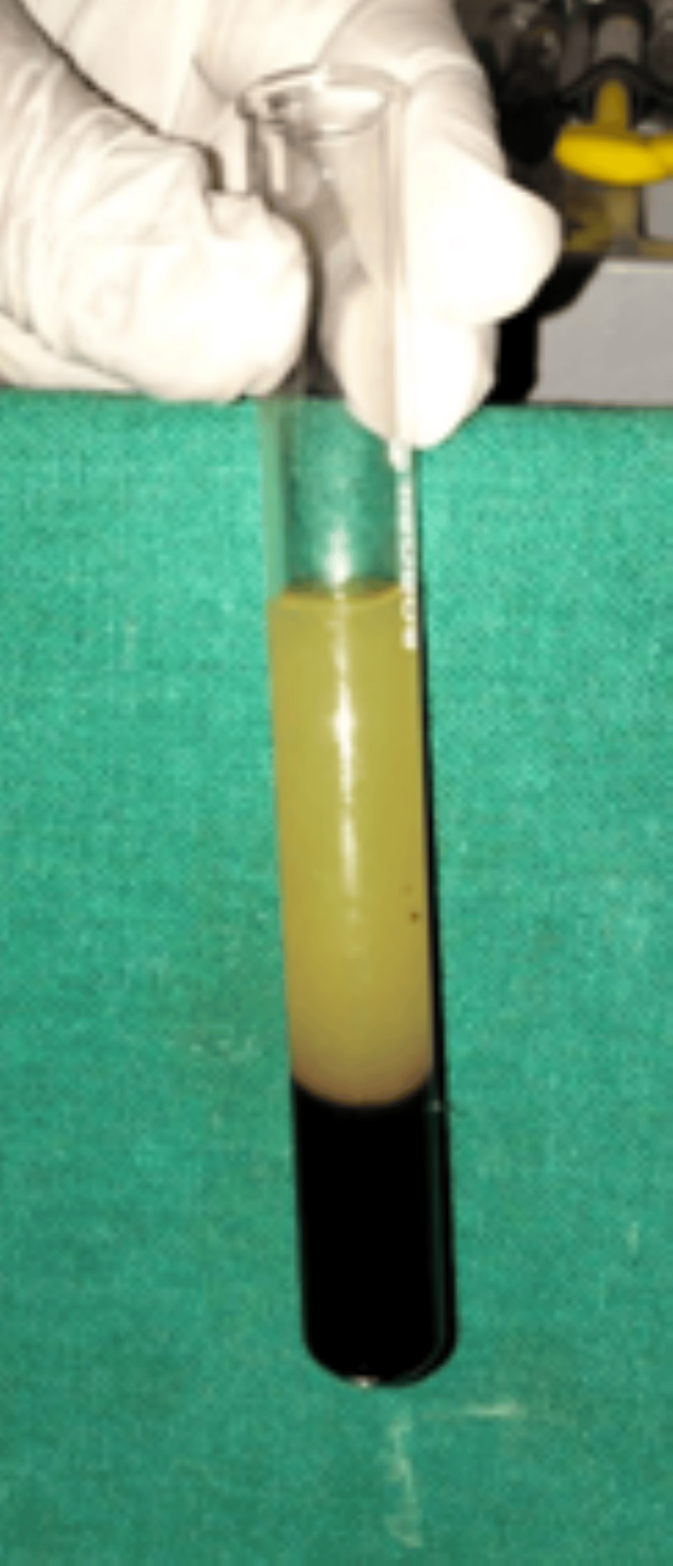
Preparation of platelet-rich fibrin.

The avulsed teeth were repositioned along with platelet-rich fibrin (PRF) into the socket (Figure 5).

**Figure 6:**
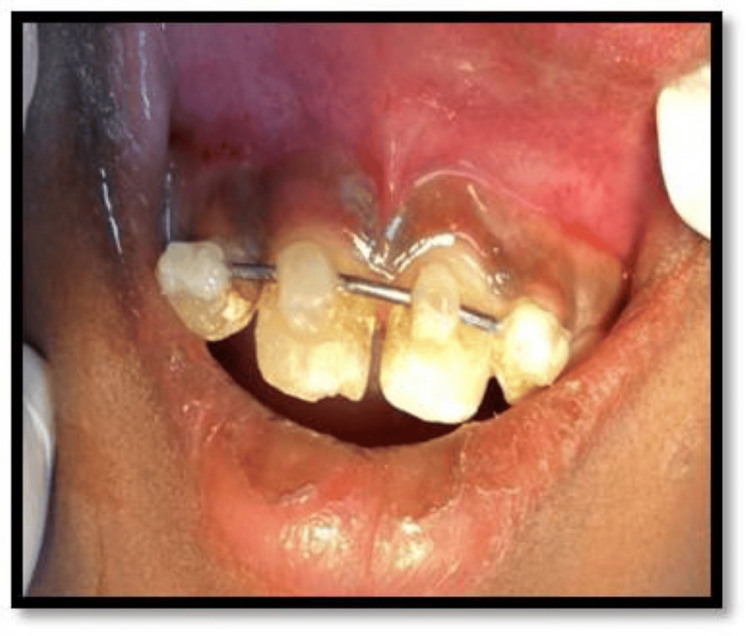
Reimplanted teeth splinted with composite resin.

Following the reimplantation of teeth into the socket, teeth were splinted with orthodontic wire and composite resin (Figure 6).

**Figure 7:**
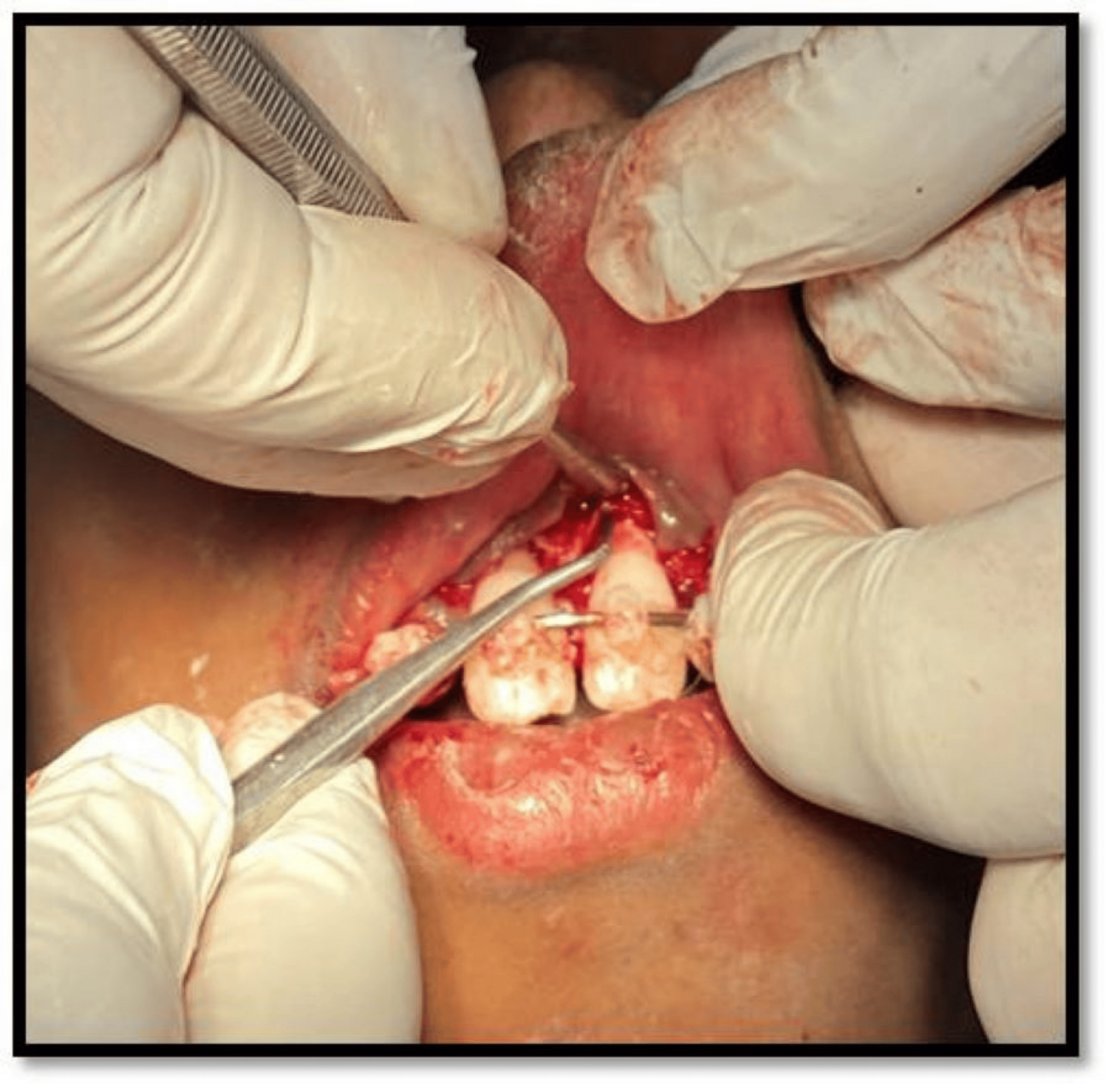
Placement of simvastatin along with platelet-rich fibrin after labial flap elevation.

The child was prescribed prophylactic antibiotic therapy and an analgesic for five days. On the second recall, grade 3 and grade 2 mobility was noticed with probing depth of 9 mm and 5 mm in 11 and 21, respectively. Upon radiographic examination, severe bone loss was noticed. Access opening was done and working length was determined using Ingle’s method. The root canal space was obturated with calcium hydroxide with iodoform paste. The gingival flap near teeth 11 and 21 was raised to remove granulation tissue between the root surface and bone. Root surface conditioning was done with 17% ethylenediaminetetraacetic acid. A combination of bone graft and simvastatin powder was applied along the PRF membrane to further inhibit bone resorption (Figure 7).

**Figure 8:**
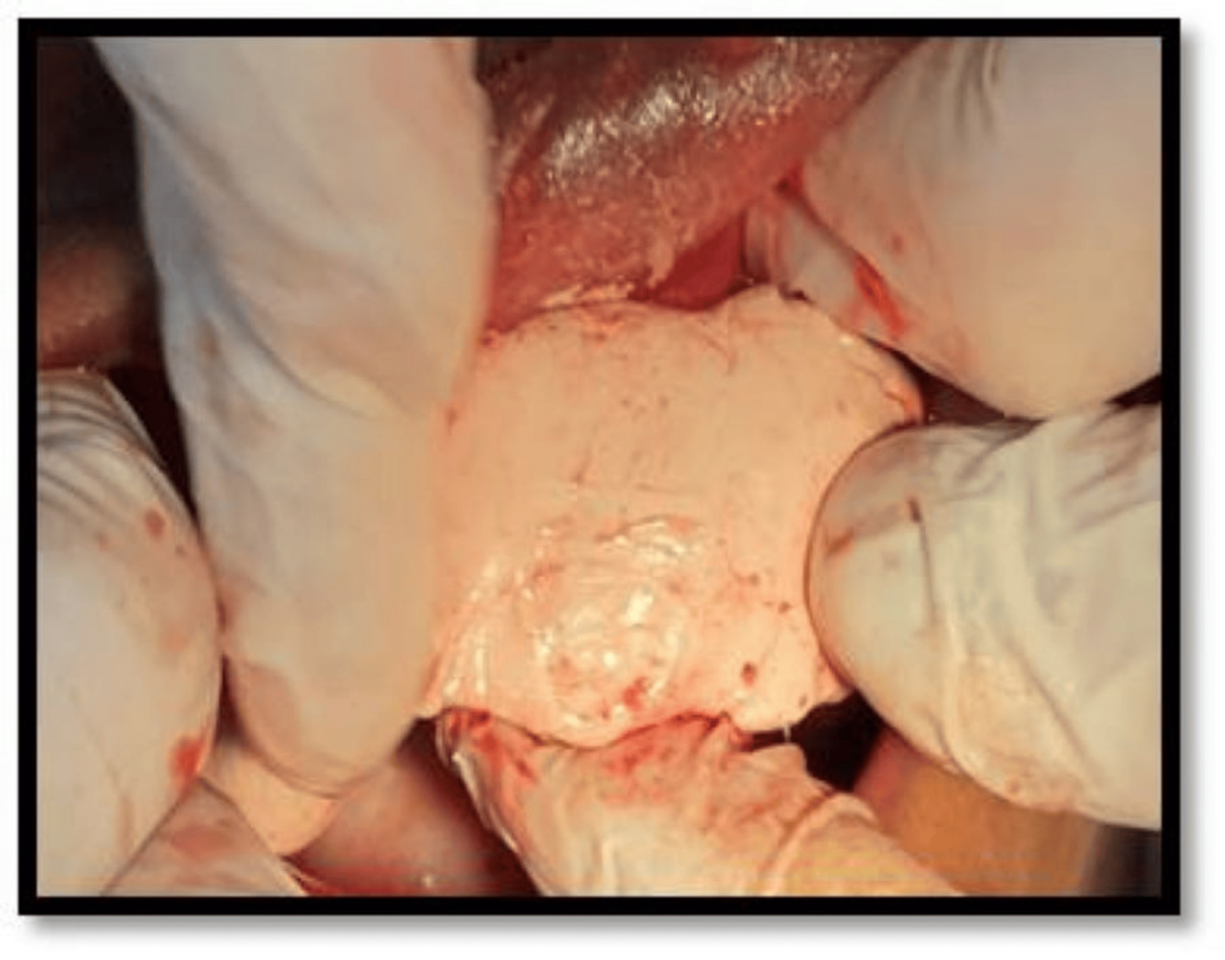
Placement of periodontal pack after flap closure.

Sutures were placed and a periodontal pack was administered (Figure 8).

**Figure 9:**
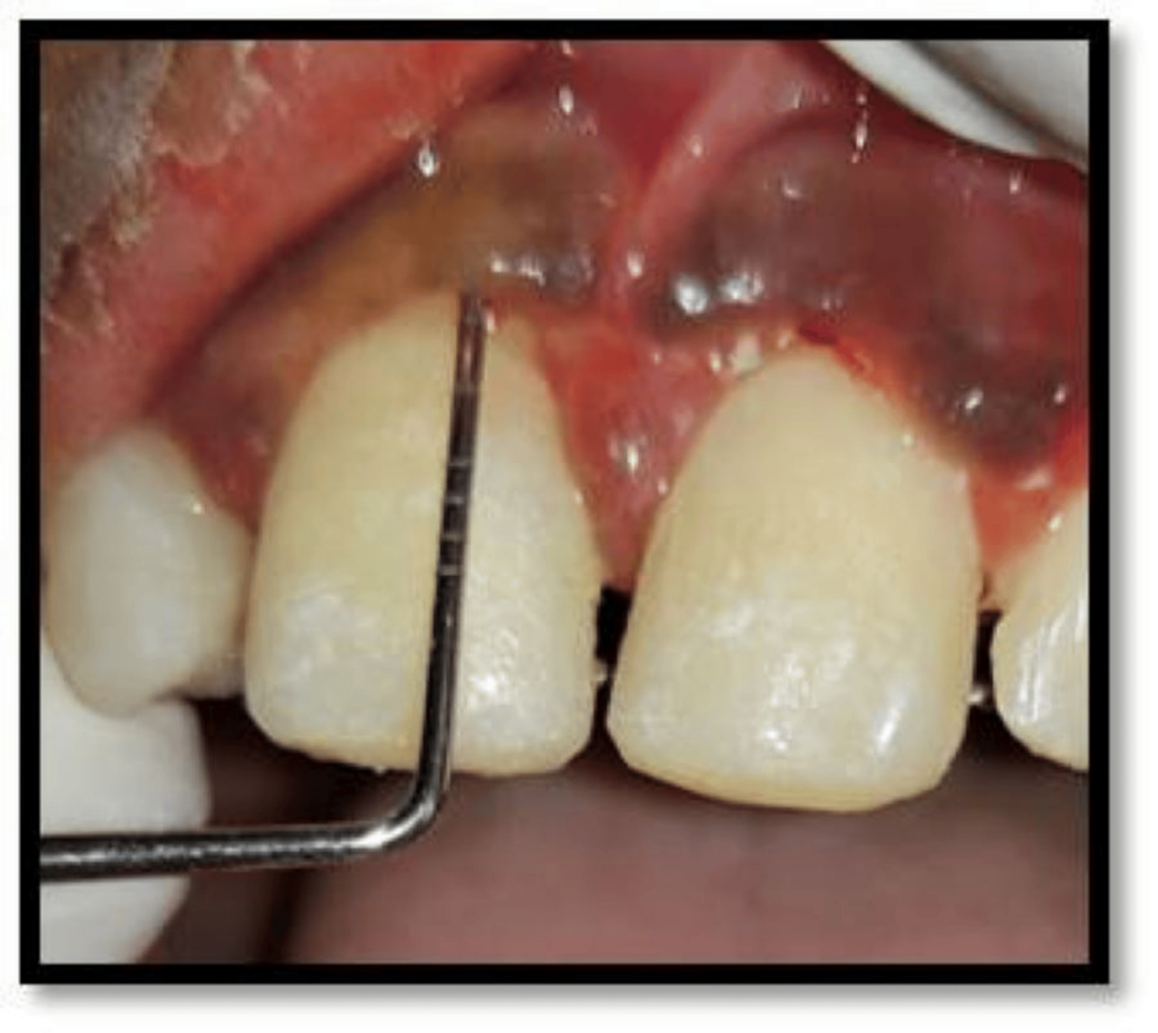
Probing depth of 3 mm in tooth 11 on the fourth recall visit.

During the third recall, probing depths of 5 mm and 3 mm were noted in 11 and 21, respectively. Mineral trioxide aggregate plus an apical plug was placed apically followed by gutta-percha obturation. On the fourth recall, the splint was removed, and the probing depth was 3 mm for both 11 and 21 (Figure 9).

**Figure 10:**
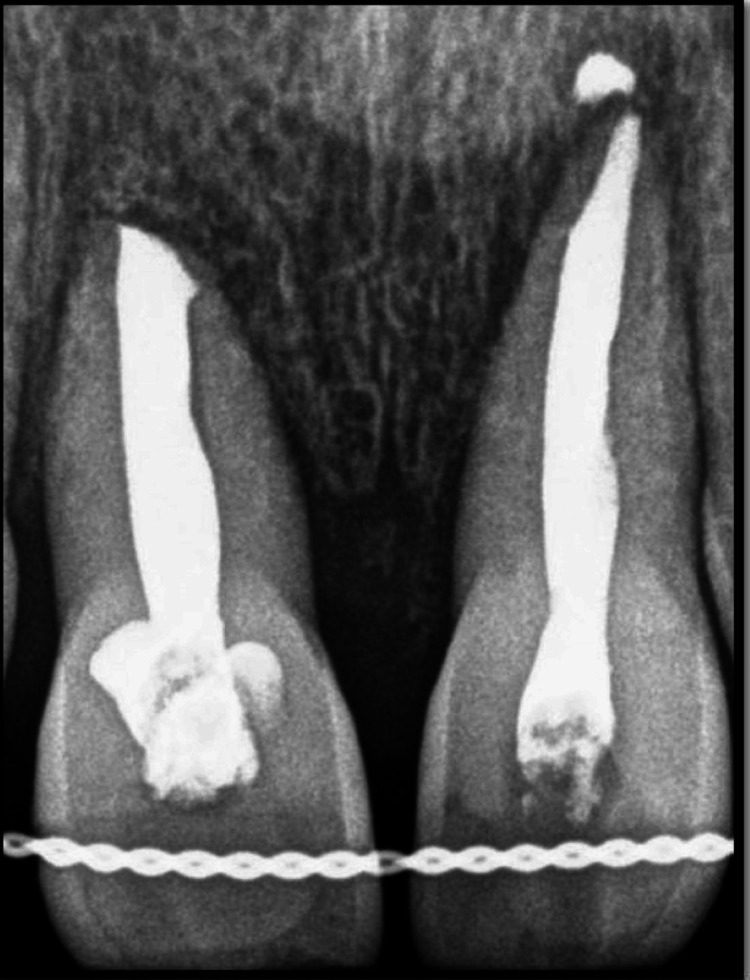
Postoperative radiograph after a one-year follow-up.

On the follow-up visit after one year, a radiograph was obtained, as shown in Figure 10.

Case 2

**Figure 11:**
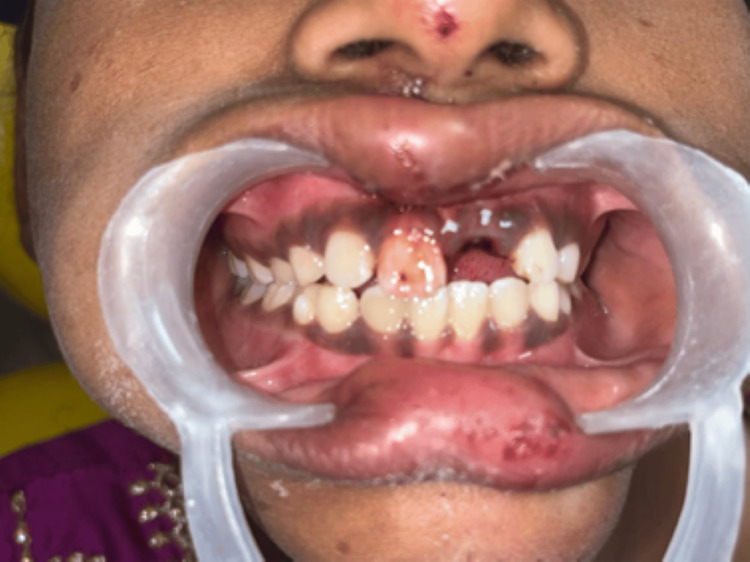
Preoperative image showing empty tooth socket following avulsion of 21.

An 11-year-old girl presented to the department seeking care for an avulsed tooth. The trauma occurred 26 hours ago due to a fall from a vehicle. The avulsed tooth was recovered and stored in dry conditions. The medical history was inconsequential. An intraoral examination detected a blood clot in the dental socket of the upper left permanent central incisor (tooth 21) (Figure 11).

**Figure 12:**
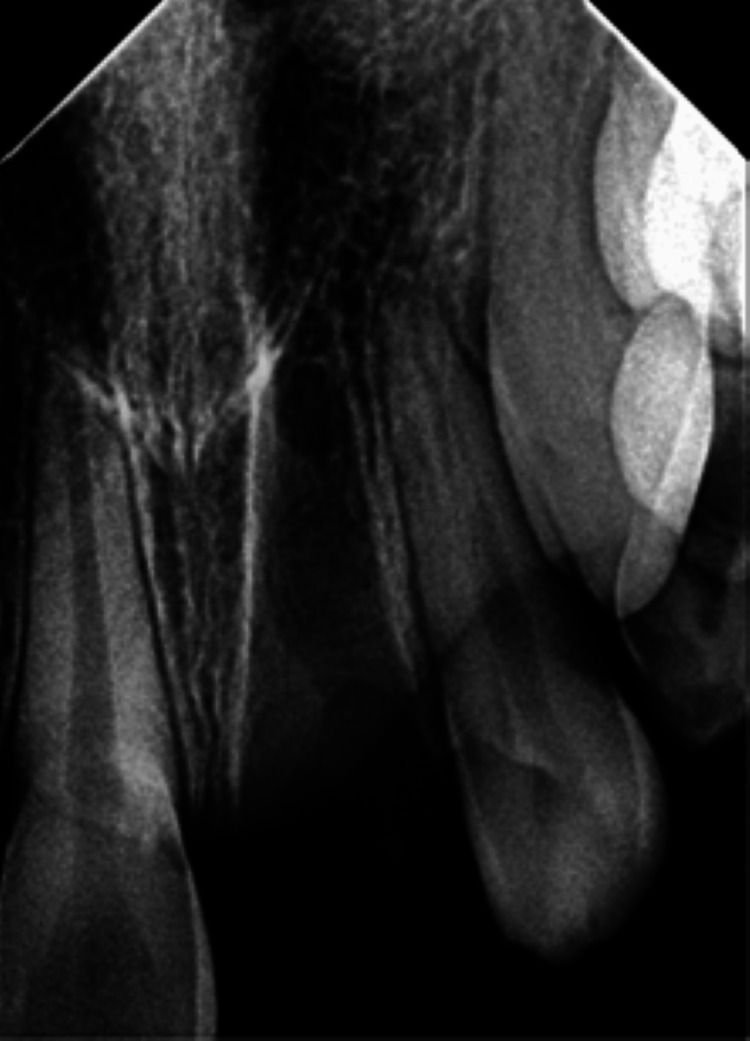
Preoperative radiograph showing empty tooth socket of tooth 21 following an avulsion injury.

The intraoral periapical radiograph revealed an empty alveolar socket of 21 (Figure 12).

**Figure 13:**
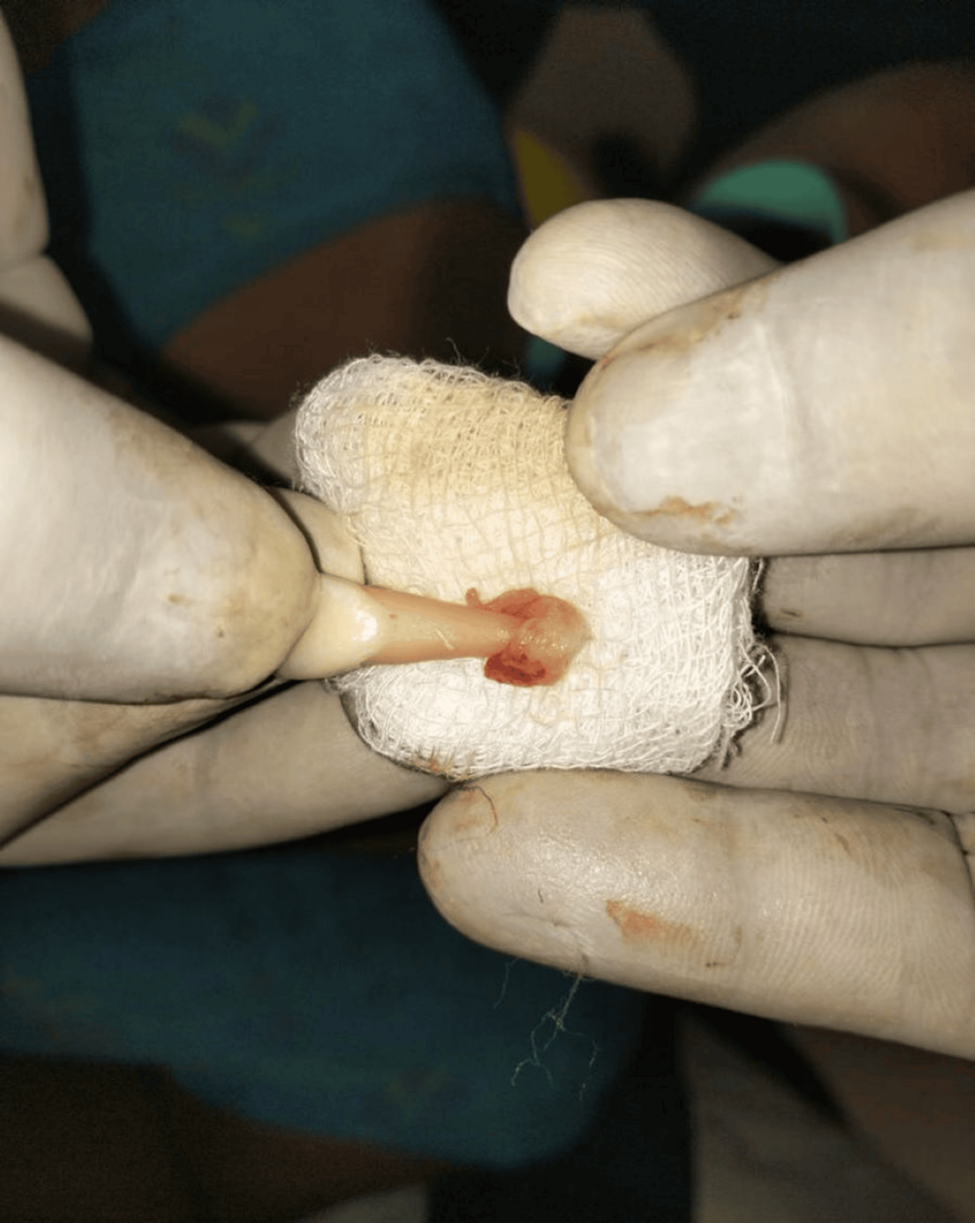
Platelet-rich fibrin placement on avulsed tooth before reimplantation.

**Figure 14:**
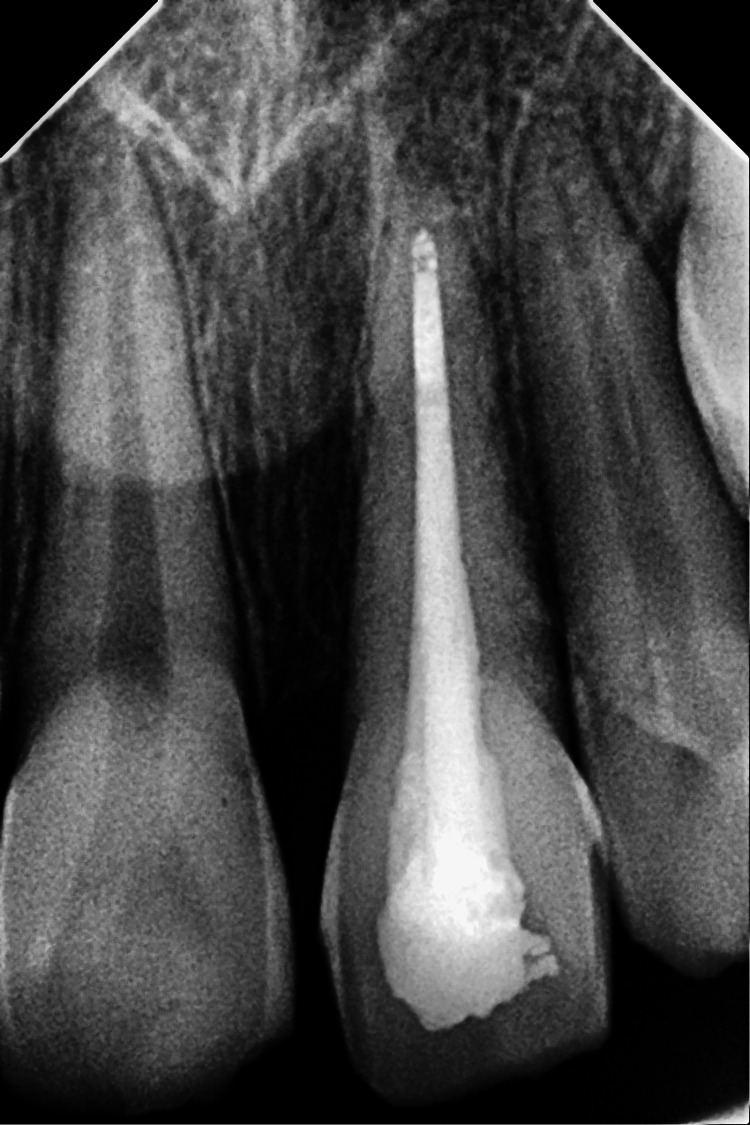
Postoperative radiograph after gutta-percha obturation.

Written informed consent was obtained from the parents after explaining the treatment plan and prognosis of the tooth. A similar procedure (Figure 13) was followed as mentioned above and a probing depth of 5 mm was observed on the second recall and 2 mm on the third recall After a one-year follow-up, a radiograph was obtained, as shown in Figure 14.

Case 3

An eight-year-old boy presented to the department with the loss of the upper front tooth. The avulsed tooth was recovered and stored in dry conditions for 24 hours. The medical history was non-significant. Intraoral examination and radiograph revealed an empty dental socket of the upper right permanent central incisor (tooth 11). After obtaining written informed consent from the parents, the treatment was performed in a manner similar to the above-mentioned cases. On the second recall, a probing depth of 6 mm was observed, and a probing depth of 2 mm was observed on the third recall. After one year, a follow-up radiograph was obtained.

Case 4

**Figure 15:**
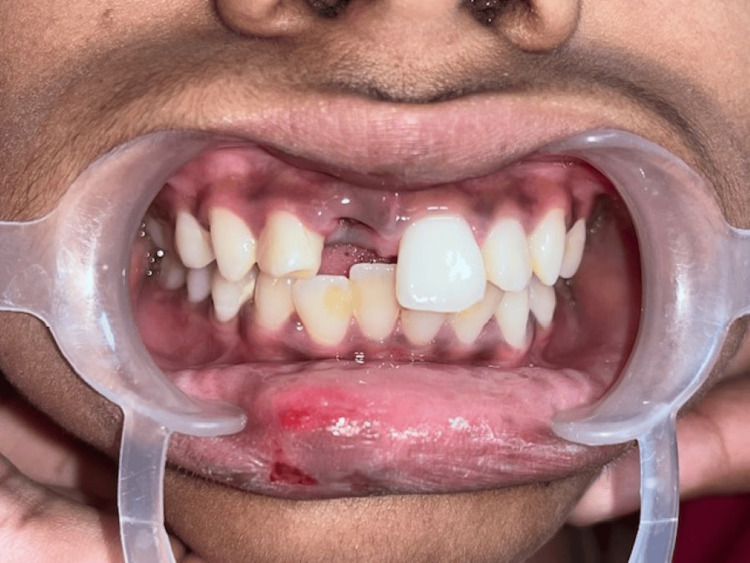
Preoperative image showing missing 11 following an avulsion injury.

A 13-year-old boy reported to the department with the loss of an upper front tooth (Figure 15).

**Figure 16:**
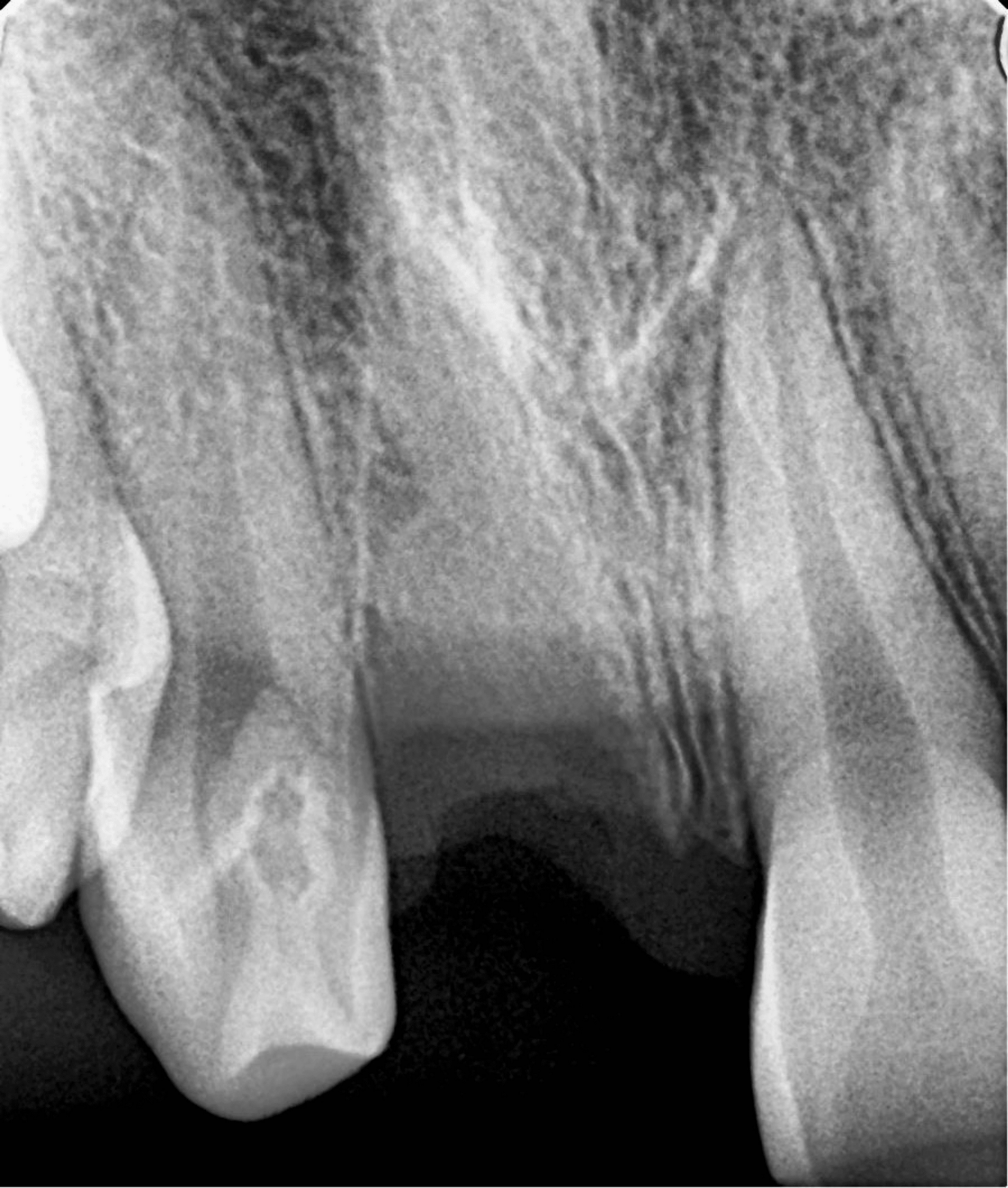
Preoperative radiograph showing empty tooth socket of tooth 11 following an avulsion injury.

The trauma occurred 96 hours ago due to a fall from a vehicle. The avulsed tooth was recovered and stored in dry conditions. The medical history was non-contributory. An intraoral examination detected a blood clot in the dental socket of the right-left permanent central incisor (tooth 11). The intraoral periapical radiograph revealed an empty alveolar socket of 11 (Figure 16).

**Figure 17:**
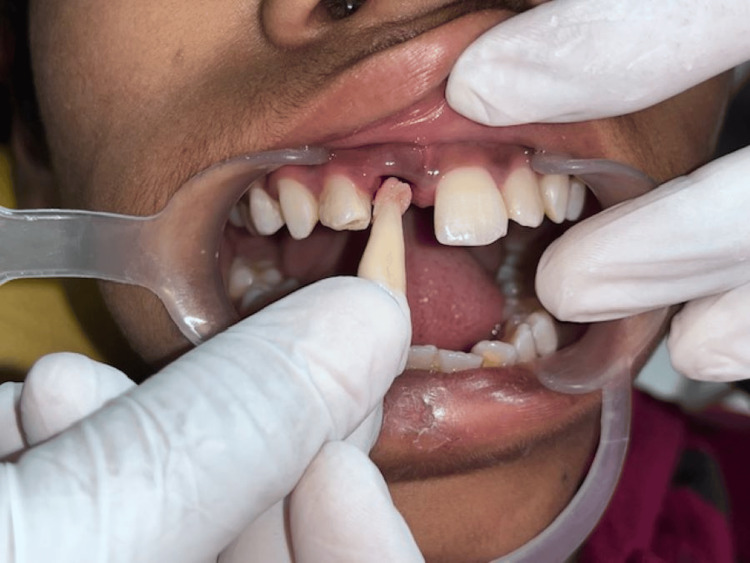
Reimplantation of avulsed tooth into the socket along with platelet-rich fibrin.

**Figure 18:**
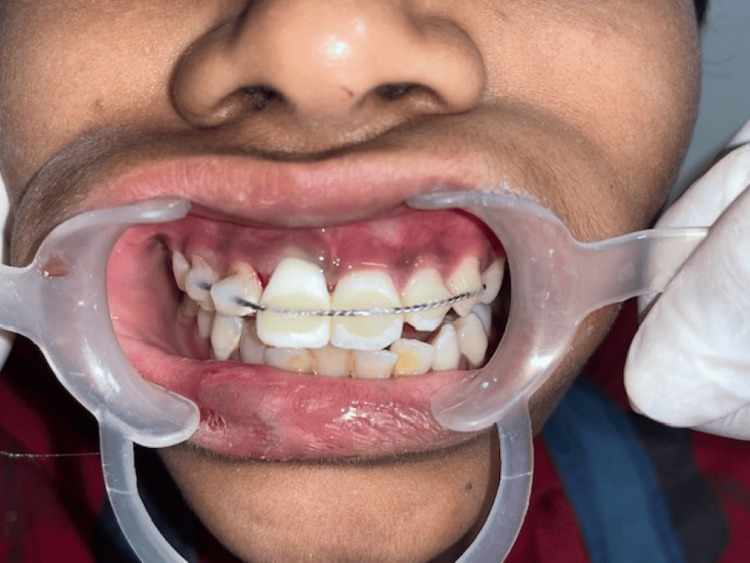
Composite splinting after the reimplantation of the avulsed tooth into the socket.

**Figure 19:**
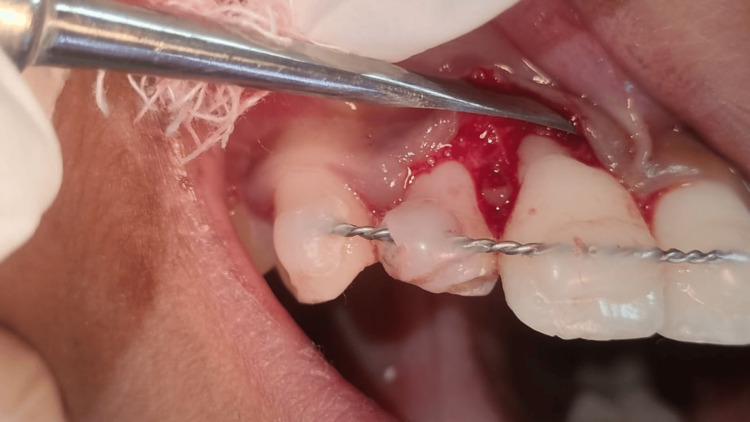
Application of simvastatin as a graft material after reflecting the flap.

Written informed consent was obtained from the parents after explaining the treatment plan and prognosis of the tooth. A similar procedure was followed, as shown in Figures 17-19.

**Figure 20:**
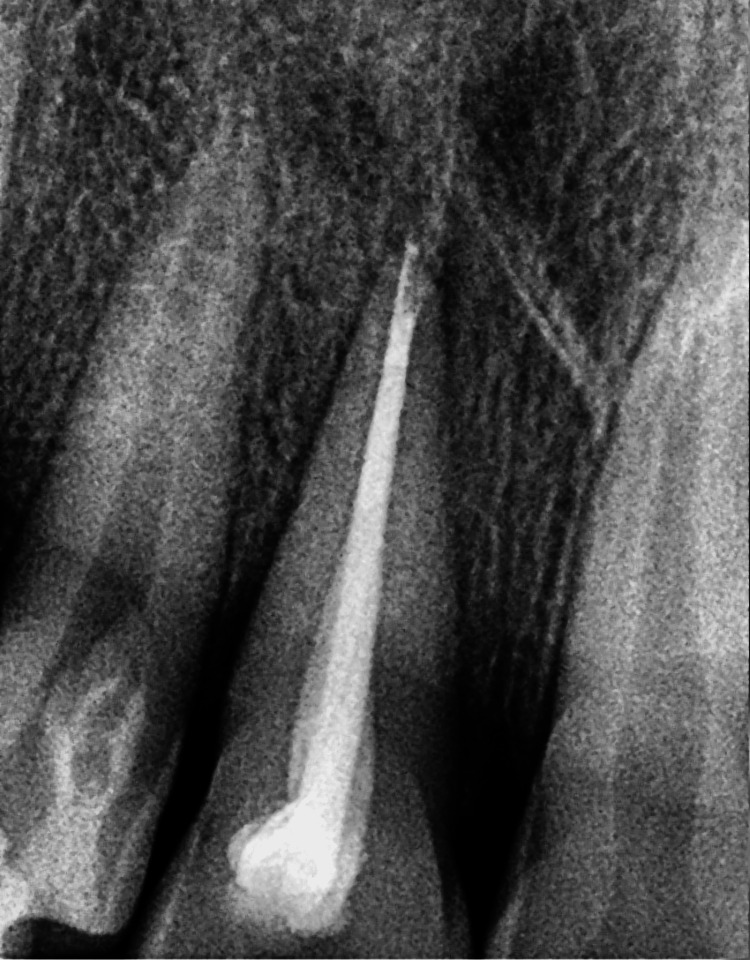
Postoperative radiograph after a one-year follow-up.

A probing depth of 6 mm was observed on the second recall and 2 mm on the third recall. After a one-year follow-up, a radiograph was obtained, as shown in Figure 20.

## Discussion

Management of avulsion is challenging for dentists. The significance of storage media and the duration of extraoral dry time have been recognized as pivotal factors in influencing treatment outcomes, as these conditions are crucial for preserving the viability of cells, particularly those in the periodontal ligament, which are essential for tooth reattachment. The reported cases of successful reimplantation with an increased extraoral dry time have adopted the principles of treating the root surface and various components of supporting tissues (periodontium) [12].

In an ideal healthy environment, the presence of the cementum and predentin act as a barrier preventing the infiltration of the cells. The balance between formation and resorption is maintained in equilibrium by the surrounding structures by a process known as coupling [13]. Any damage to surrounding structures leads to the infiltration of clastic cells, thereby initiating the resorptive process.

The severity of root resorption in a reimplanted tooth depends on the extent and intensity of the injury and the persistence of the stimulus. Hence, guidelines have been formulated that if dry time is more than one hour the surface has to be debrided to eliminate non-viable periodontal ligament fibers, as they may serve as a conduit for contamination and infection leading to inflammatory root resorption [14]. Additionally, undifferentiated mesenchymal cells in the socket may be triggered by non-viable periodontal tissue, differentiating into osteoblasts and causing replacement resorption or ankylosis [13]. Therefore, surface treatment of the avulsed tooth is pivotal to the success of delayed replantation in preventing problems including inflammatory/replacement root resorption [15].

Agents such as sodium hypochlorite solution, glucosaline solution, saline solution, 2% sodium fluoride solution, 12% chlorhexidine, citric acid, 2% APF gel, 10% phosphoric acid, metronidazole solution, hydrofluoric acid gel, doxycycline solution, and betadine are suggested to disinfect and create a root surface resistant to resorption [16-28]. Various agents have been used for root conditioning (Table 1).

**Table 1:** Agents used in disinfecting the root surface .

Authors	Extraoral dry time	Agent	Total follow-up	Outcomes
Suresh (2021) [16]	More than 4 days	Saline followed by betadine	1 year	No complications
Brunet-Llobet et al. (2018) [17]	26 hours	Saline	10 years	Ankylosis and root resorption of the transplanted tooth occurred and the teeth were ultimately addressed cosmetically. The clinical and radiological results after 10 years showed a satisfactory functional outcome
Vafaei et al. (2018) [18]	75 hours	Saline followed by 2% sodium fluoride for 20 minutes	3 years	No complications
Daga et al. (2018) [19]	72 hours	Saline solution, 2% sodium fluoride solution, doxycycline antibiotic solution	1 year	No complications
Ines and Nabiha (2016) [20]	24 hours	Isotonic saline solution, 12% chlorhexidine	1 year	Prolonged dry conditions of the avulsed tooth and rigid splinting may explain rapid complete root resorption. There was dentoalveolar ankylosis associated with osseous replacement, i.e., replacement resorption, which was followed by tooth extraction
Agarwal et al. (2016) [21]	30 hours	Saline irrigation, 2.4% acidulated sodium fluoride solution	1 year	No complications
Rahbar and Hassani Dehkharghani (2016) [22]	30 hours	Hydrofluoric acid gel, doxycycline solution	1 year	No complications
Harris et al. (2014) [23]	36 hours	2.5% NaOCl for 20 minutes, citric acid for five minutes, followed by 2% acidulated phosphate fluoride for 5 minutes	24 months	No complications
Anand et al. (2014) [24]	24 hours	10% phosphoric acid, acidulated phosphate fluoride gel, metronidazole solution	1 year	After 12 weeks, the tooth developed mild infraocclusion (of about 1 mm) and progressive ankylosis but remained functional and was aesthetically acceptable
Ize-Iyamu and Saheeb (2013) [25]	72 hours, 72 hours	NaOCl solution for 15 minutes	12 weeks, 16 months	Ankylosis and root resorption of the transplanted tooth occurred. No Complications
Ritwik et al. (2012) [26]	42 hours	Doxycycline soak, fluoride soak	7 months	Root resorption of the reimplanted tooth occurred
Cobankara and Ungor (2007) [27]	7 days	2.5% NaOCl solution	8 years	After 8 years, there was a pink appearance on the cervical buccal surface of the replanted tooth because of progressive replacement resorption
Duggal et al (1994) [28]	10 days	2.4% sodium fluoride	28 months	Root resorption of the reimplanted tooth occurred

These agents only disinfect the root surface but fail to promote the growth of tissue on the alveolar side. Hence, clinical failure has been reported over a duration of time.

In this case series, we debrided the root surface with scalar followed by treating the root surface with APF gel. Calcium hydroxide with iodoform in the root canal slowly releases calcium ions, preventing infection and inflammation from the canal. In our cases, a week after replantation, endodontic therapy was started, and then calcium hydroxide with iodoform paste was placed in the root canal for a week. With its antibacterial properties, ability to block bacterial enzymes, activation of tissue enzymes such as alkaline phosphatase, and stimulation of mineralization, calcium hydroxide with iodoform paste aids in comprehensive cleaning and lowers the likelihood of root resorption associated with replanting [29]. While the current standards advocate for a four-week placement duration for calcium hydroxide with iodoform paste, shorter placement times have been demonstrated to be equally effective in the absence of pathology [30]. This step can only prevent resorption of the tooth and does not promote an altered healing process.

The possibility of survival of periodontal cells and other cells inside the alveolus and the ability of these to start endogenous repairing mechanisms such as cell-based therapy or agents that induce activation of stem cells has gained importance over the past few years [31]. The presence of new inflammatory mediators in delayed reimplantation has raised the need for new studies and approaches in this direction [32]. Therefore, modulation of immune response along with endogenous repair could be a viable option in delayed reimplantation cases.

Emdogain (enamel matrix protein) was able to stimulate the formation of new periodontal ligament cells from bone marrow progenitor cells [33,34]. It has also been proven in experimental conditions within a stipulated dry time of over 60 minutes. It may not be useful in the out-of-time range but can be considered fairly with the above-mentioned dry time. Alendronate was found to have similar resorption slowing effects as fluoride when used topically but further studies need to be conducted to evaluate whether its effectiveness is superior to fluoride and whether this justifies its added cost [35].

PRF consists of a high number of growth factors that stimulate cell migration, proliferation, and differentiation [36]. PRF shows a prolonged slow release of growth factors demonstrating a longer duration of biological effects. Furthermore, teeth treated with PRF alone show the formation of tissue similar to the periodontal ligament, which may be explained by the presence of stem cells in the periodontal tissue remaining in the alveolus which can differentiate and form tissue similar to the periodontal ligament [37]. Seo et al. reported the multipotent ability of periodontal ligament stem cells isolated from human-impacted third molars to differentiate into periodontal ligaments, alveolar bone, cementum, peripheral nerves, and blood vessels; therefore, it can be assumed that stimulation of periodontal tissue stem cells by PRF can lead to better healing of an injury of the periodontal ligament and associated structures [38].

Other studies have reported that PRF can stimulate the proliferation capacity of human dental pulp cells, osteoblasts, oral bone mesenchymal stem cells (MSCs), gingival fibroblasts, and periodontal ligament cells, but not epithelial cells [39]. The underlying mechanism of this stimulation effect is the high quantity of growth factors contained in a well-prepared PRF. Dohan et al. found that PRF stimulated the proliferation of human oral bone MSCs in a dose-dependent manner which could be a drawback in treating delayed reimplantation cases as PRF alone cannot hold the inflammatory process. It needs an adjunct that has similar properties and has a cumulative effect [40]. To fasten healing a material should have a synergistic action with PRF. Some agents that show similar patterns of repair cell-based periodontal regeneration as PRF are not clinically viable [31].

Statins have similar properties compared to PRF. Statins, widely known as competitive inhibitors of 3-hydroxy-3-methylglutaryl coenzyme A (HMG-CoA) reductase, lower cholesterol levels and have been shown to increase bone morphogenetic protein 2 (BMP-2)-induced bone formation. Of these, simvastatin use has yielded similar and good results [41]. Simvastatin shows pleiotropic effects such as anti-inflammatory, antimicrobial, bone regeneration, bone tissue engineering, dentinogenesis, angiogenesis, and odontoblastic differentiation [42-44]. The following positive effects of simvastatin have been reported in the literature: it jump-starts the cascade of osteogenesis in the bone graft, improves trabecular bone density, provides earlier availability of growth factors and BMP, promotes early consolidation of the graft, hastens the mineralization of the graft, enhances bone regeneration, activates wound healing [45].

In a systematic review of the effects of statins, simvastatin was proven to significantly reduce the expressions of IL-6, IL-8, IL-1β, and tumor necrosis factor-α levels. The spectrum activity of these drugs against Gram-positive and Gram-negative bacteria has also been reported. These drugs also show in vitro activity against Gram-positive and Gram-negative bacteria. A vital point to consider for selecting the drug in delayed reimplantation cases is its ability to significantly improve reduction in periodontal depth, increase clinical attachment levels, and bone regeneration [46].

Simvastatin when mixed with hydroxyapatite has shown promising results in bone formation [47]. Similarly, PRF-mixed hydroxyapatite crystals have also shown promising results [48]. The mixture of simvastatin with hydroxyapatite along with PRF application was able to control the post-reimplantation reaction after one week and was able to form bone around the tooth. The role of PRF is to hold the hydroxyapatite crystals and simvastatin for prolonged release which restricts inflammatory mediators, fastens the healing process, stimulates the transformation of stem cells into respective cells, and increases the pace of bone formation. These processes occur synchronously which led to a better prognosis in our cases.

In all the cases of delayed reimplantation, a reaction of surrounding alveolar tissue was observed within the first week of reimplantation. This could be an inflammatory reaction or a reaction of the tissues to microbes present on the root surface. PRF placed in a few of our cases also showed similar effects. Hence, PRF is minimally effective or not effective alone. Therefore, the addition of simvastatin changed the outcome due to its synergistic action.

The combination of these drugs might act on either side of the tissues at a considerable pace, i.e., promoting bone formation as well as formation of periodontium around the tooth may be key for the success in delayed reimplantation of the tooth.

## Conclusions

The success and failure of delayed teeth reimplantation depend on multiple factors. A single or two protocols may not lead to positive results, requiring a combination of various treatment measures. Combining multiple protocols can be a key step in treating these conditions. A drawback of this case series is that the review of cases was limited to one to one and a half years. A randomized controlled trial and long-term studies are required to evaluate and calibrate the dose and ratio of the agents used in this study.
